# Predicting Anticancer Drug Responses Using a Dual-Layer Integrated Cell Line-Drug Network Model

**DOI:** 10.1371/journal.pcbi.1004498

**Published:** 2015-09-29

**Authors:** Naiqian Zhang, Haiyun Wang, Yun Fang, Jun Wang, Xiaoqi Zheng, X. Shirley Liu

**Affiliations:** 1 Department of Mathematics, Shanghai Normal University, Shanghai, China; 2 Department of Bioinformatics, School of Life Science and Technology, Tongji University, Shanghai, China; 3 Department of Biostatistics and Computational Biology, Dana-Farber Cancer Institute and Harvard School of Public Health, Boston, Massachusetts, United States of America; 4 Center for Functional Cancer Epigenetics, Dana-Farber Cancer Institute, Boston, Massachusetts, United States of America; Memorial Sloan-Kettering Cancer Center, UNITED STATES

## Abstract

The ability to predict the response of a cancer patient to a therapeutic agent is a major goal in modern oncology that should ultimately lead to personalized treatment. Existing approaches to predicting drug sensitivity rely primarily on profiling of cancer cell line panels that have been treated with different drugs and selecting genomic or functional genomic features to regress or classify the drug response. Here, we propose a dual-layer integrated cell line-drug network model, which uses both cell line similarity network (CSN) data and drug similarity network (DSN) data to predict the drug response of a given cell line using a weighted model. Using the Cancer Cell Line Encyclopedia (CCLE) and Cancer Genome Project (CGP) studies as benchmark datasets, our single-layer model with CSN or DSN and only a single parameter achieved a prediction performance comparable to the previously generated elastic net model. When using the dual-layer model integrating both CSN and DSN, our predicted response reached a 0.6 Pearson correlation coefficient with observed responses for most drugs, which is significantly better than the previous results using the elastic net model. We have also applied the dual-layer cell line-drug integrated network model to fill in the missing drug response values in the CGP dataset. Even though the dual-layer integrated cell line-drug network model does not specifically model mutation information, it correctly predicted that BRAF mutant cell lines would be more sensitive than BRAF wild-type cell lines to three MEK1/2 inhibitors tested.

## Author Summary

In this study, using the Cancer Cell Line Encyclopedia (CCLE) and Cancer Genome Project (CGP) studies as benchmark datasets, we explored the application of similarity information between cell lines and drugs in drug response prediction. We found that similar cell lines by gene expression profiles exhibit similar response to the same drug. Meanwhile, drugs with similar chemical structures also show similar inhibitory effects across different cell lines. Based on the above observations, we proposed a dual-layer network and local weighted model to predict drug response of a cell line using proximal information of the drug-cell line network. The only three parameters of our model are optimized by leave-one-out cross-validation for each drug. Two case studies of MAPK and ERK signal pathways on CCLE dataset proved that the predicted-to-observed correlations of our dual-layer network model is significantly better than the previous predictor using elastic net model. Interestingly, predictions based on drug similarity network (DSN) alone were much better than those based on cell line similarity network (CSN) alone for most drugs, implying that drug similarities are more informative for drug response prediction than cell line similarities. Our network model can be applied to predict the response of a new cell line to existing already tested drugs or to predict the response of an existing cell line to new drugs, thus potentially saving the cost in a drug-cell line screening.

## Introduction

Over the past two decades, substantial improvements in high-throughput profiling technologies and systems approaches have increased expectations that personalized or precision medicine will become the paradigm of future medical science [1–3]. In contrast to the one-size-fits-all approach that has dominated cytotoxic chemotherapy, personalized medicine exploits tumor response and vulnerability based on identified molecular traits to overcome some of the limitations associated with conventional symptoms-oriented disease diagnoses and therapies. The most important step in implementing personalized medicine will be the identification of biomarkers useful for predicting the drug response of a given patient [4–6]. However, the development of predictive biomarkers would require substantial efforts and is often prohibitively expensive in human or animal models. Therefore, many studies conduct large-scale drug screenings on cultured human cell line panels to identify predictive biomarkers [7]. One of the earliest such attempts is the NCI-60 study [8,9], which included a set of 60 human cell lines and their responses to more than 100,000 chemical compounds. Drug response results for the NCI-60 dataset [10,11] revealed that different types of cancers have different drug response signatures, and that different tumors derived from the same type of cancer may have distinct molecular patterns [12].

Two recent consortiums, the Cancer Cell Line Encyclopedia (CCLE) [13] and Cancer Genome Project (CGP) [14], systematically addressed the issue of predictive biomarker identification by collectively analyzing around 1,000 clinically-relevant human cell lines and their pharmacological profiles for 149 cancer drugs. These two studies also included the gene expression profiles and mutation status for each cell line, and applied the elastic net model to select expression and mutation signatures that are predictive of drug responses. Based on the same dataset, Geeleher et al. applied another sparse regression model, Ridge, to predict drug response for breast cancer cell lines using baseline gene expression data [15]. Brubaker et al. used a probabilistic graphical model named PARADIGM to infer patient-specific genetic activity by integrating copy number and gene expression data into a factor graph model [16]. Menden et al. integrated genomic features of cell lines (mutation, copy number and microsatellite instability) with chemical properties of drugs to represent each cell line-drug pair, and used neural network to predict drug response in CGP dataset [17]. Ammad-ud-din et al. proposed a kernelized Bayesian matrix factorization model to integrated drug property matrix and cell line genomic properties matrix [18]. This kind of approach could capture the nonlinear relationships between drug response and chemical descriptors (or cell line genomic features) by a kernel strategy and thus was adopted in many other areas including drug-target interaction prediction [19]. Despite achieving promising results for certain drugs, these approaches do not take into consideration two important characteristics of a cancer cell drug response screens, including: 1) genetically similar cell lines or samples may also respond very similarly to a given drug; and 2) structurally related drugs may have similar therapeutic effects due to their shared molecular structure or targeting patterns. Incorporating similarities between cell lines and drugs could potentially improve the drug response prediction.

In this study, motivated by the integrated model in disease genes prioritization [20,21], we constructed a dual-layer integrated cell line-drug network, and modeled the integrated similarity between cell lines based on their gene expression profiles, and between drugs based on their 1-D and 2-D chemical structures. We predicted the response of a given cell line to a drug based on a weighted model using either one or both layers of the cell line similarity network (CSN) and the drug similarity network (DSN). Our proposed dual-layer integrated cell line-drug network model combines the predictions from the individual CSN and DSN layers, and predicts a response of a cell line to a drug based on how similar cell lines (CSN) respond to similar drugs (DSN). Instead of selecting a large number of genomic features from previous studies to predict tumor drug responses, our model uses only three parameters to build a prediction model to decrease the risk of overfitting. Using CCLE and CGP studies as benchmark datasets, we evaluated the predictive power of our model and found that our dual-layer integrated cell line-drug network model is significantly better than model that use either the CSN or the DSN layer alone, as well as the elastic net model. We also applied the dual-layer network model to fill in all of the missing drug response values (activity area and IC50) in the CGP dataset, and found that our predicted responses to three MEK1/2 inhibitors in the CGP study show a distribution very similar to other available drug response values.

## Results

### Similar cell lines and similar drugs have similar responses

**Fig 1:**
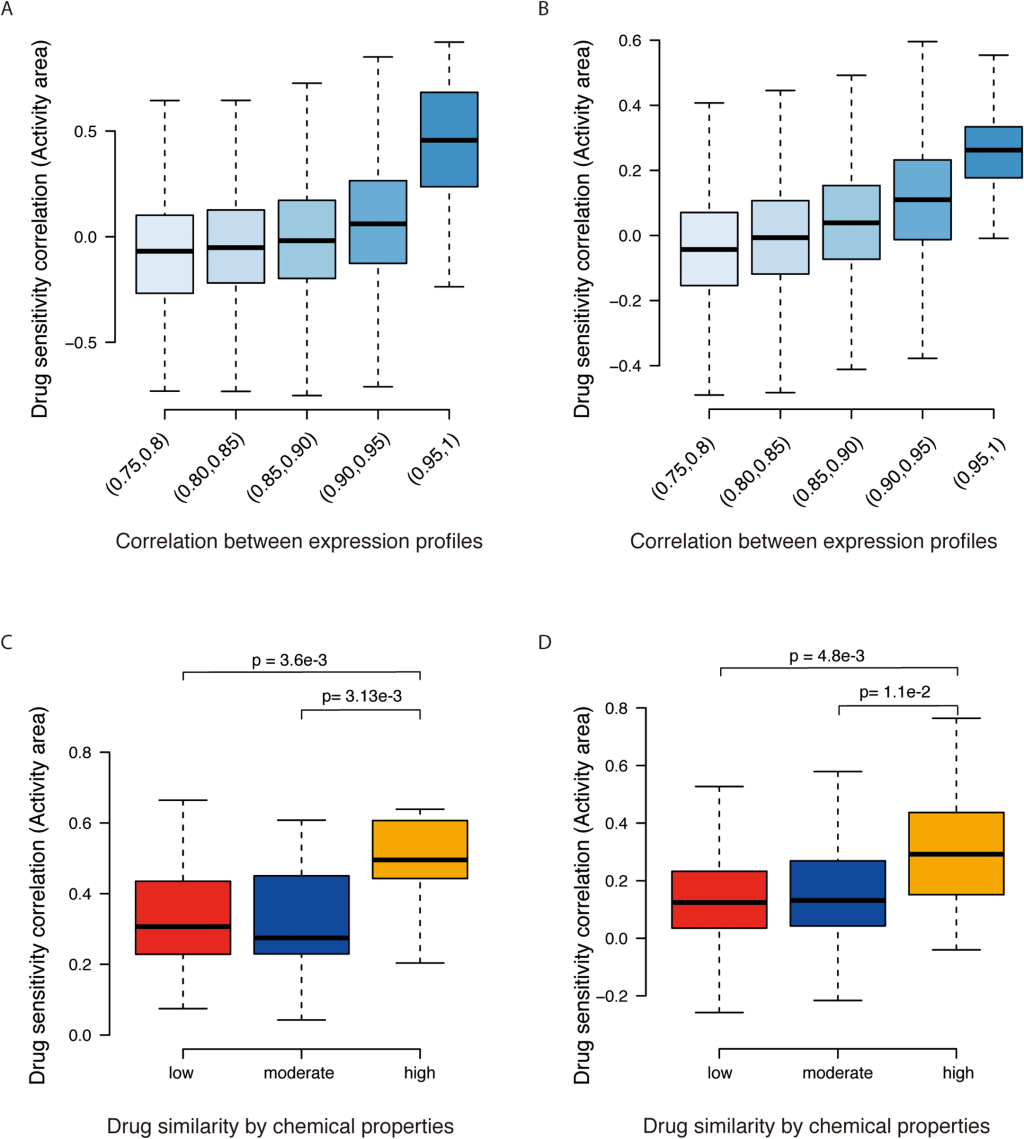
Model assumption. (A, B) Box plots showing cell lines with similar gene expression profiles responding similarly to the same drugs. The X-axis indicates the Pearson correlation coefficients between all possible cell line pairs based on expression profiles. The Y-axis shows the correlations of their drug response vectors as measured by activity area in CCLE (A) and CGP (B). (C, D) Box plots showing that drugs with similar 1-D and 2-D structural features based on PaDEL exhibiting similar effects on cell lines in the CCLE (C) and CGP (D) datasets. The X-axis represents the drug similarity categories, and the Y-axis shows the correlations of drug responses across all cell lines. Statistical differences between two groups were measured by the t-test.

We built the dual-layer integrated cell line-drug network model using large pharmacogenomics datasets from the CCLE [13] and CGP [14] studies. Experimentally determined drug responses, also referred to as drug sensitivities, which were measured as activity area and IC50 in both studies. Notably, a higher value of activity area or lower value of IC50 indicates a better sensitivity of a cell line to a given drug. We first took activity area as drug response measurement. The CCLE study, for example, contains expression profiles of 491 cancer cell lines, as well as their response to 24 drugs. Since different drugs have different baseline values and ranges, we normalized the drug response data so that different drugs have the same baseline value and range across all cell lines. We calculated the Pearson correlation of gene expression profiles and the Pearson correlation of drug responses by activity area for each cell line pair. Drug sensitivity correlations were significantly higher for cell lines with more similar gene expression profiles (Fig 1A). The CGP dataset contains response data and expression profiles for 653 cell lines treated with 139 drugs. In agreement with the CCLE observations, CGP cell lines with higher gene expression similarity show higher drug response correlations for all the drugs tested (Fig 1B). These results suggest that cell lines with similar gene expression profiles exhibit similar drug responses.

Seeing that similar cells exhibit similar drug responses, we next examined whether similar drugs have similar effects on cells. We downloaded the chemical structure files from PubChem [22] for the drugs used in the CCLE and CGP datasets, extracted the 1-D and 2-D structural features of each drug using PaDEL [23], and calculated the Pearson correlation between two drugs using these structural features. Here, 1D descriptors refer to compositional or constitutional molecular properties, and 2D descriptors include different quantitative properties of the molecular topology (see Materials and Methods for details). We divided all drug pairs into three groups according to their pairwise chemical structural similarities: low, intermediate and high. Drug pairs with more similar structures have significantly higher drug sensitivity correlations by activity area in both the CCLE (Fig 1C) and CGP (Fig 1D) datasets. This finding suggests that drugs with similar chemical structures show similar inhibitory effects across the cell lines tested. When using IC50 as drug response measurement, we observed a quite similar phenomenon in both datasets (S1 Fig).

A recent report showed inconsistent results between the CCLE and CGP datasets [24]. Our analysis above found the CSN and DSN hypotheses to be generally valid for both datasets, but the trend is a little stronger by using activity area than IC50. This might be because the activity area is measured over the whole dose-response curve, which better captures the drug effects and cell responses. In contrast, the IC50 measurement, i.e., the concentration at which the drug response reaches an absolute inhibition of 50%. So IC50 measurements only consider a single point on the dose-response curve to determine drug sensitivity for the cell lines, which might be noisier.

### Computational framework for the dual-layer integrated cell line-drug network model

**Fig 2:**
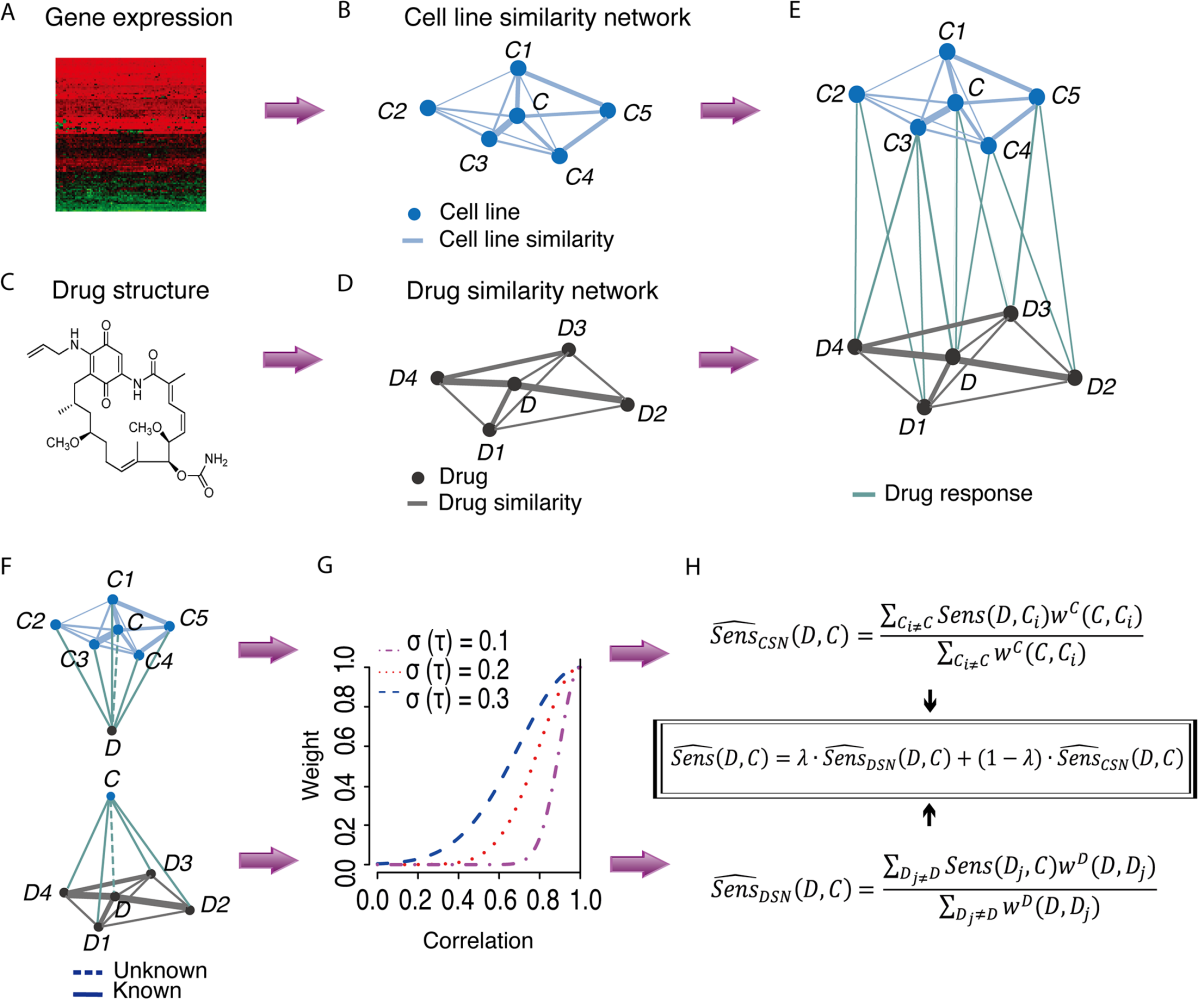
Computation framework. (A–E) Construction of the dual-layer integrated cell line-drug network model. (F–H) Drug sensitivity predictions based on information from the integrated cell line-drug network. (F) Two sub-networks for predicting the response of cell line *C* to drug *D*. (G) For each sub-network, the weighted arithmetic mean was used to measure an unknown drug-cell line response based on their neighboring cell lines or drugs. (H) Predictions from each of the two individual sub-networks were merged using a weighted model to determine the final prediction.

Based on the above results, we developed a dual-layer integrated cell line-drug network model to predict anticancer drug sensitivity using existing cancer cell line expression profiles and drug response data (Fig 2). The model integrated three types of data: 1) gene expression profiles for each cell line; 2) 1-D and 2-D chemical structural properties of each drug; and 3) the drug response for each cell line. The top layer of the network, termed cell line similarity network (CSN), predicts the response of cell line *C* to a given drug *D* using a linear model weighting drug response from cell lines with similar gene expression profiles to *C*. We calculated the gene expression correlations between *C* with all other cell lines (Fig 2A and 2B), and gave higher weights to more similar ones. The bottom layer, termed drug similarity network (DSN), predicts the response of cell line *C* to drug *D* weighting the response data on drugs similar to *D* in their chemical structures. We calculated the correlation of every drug pair based on their 1-D and 2-D chemical structure features in PubChem. The two layers, CSN and DSN, were connected using drug response data for the cell lines, which were represented as activity areas in CCLE [13] and CGP studies [14]. Notably, the network is not a complete bipartite graph, as some drug response data for some cell lines are missing in these studies, especially for the CGP dataset.

**Fig 3:**
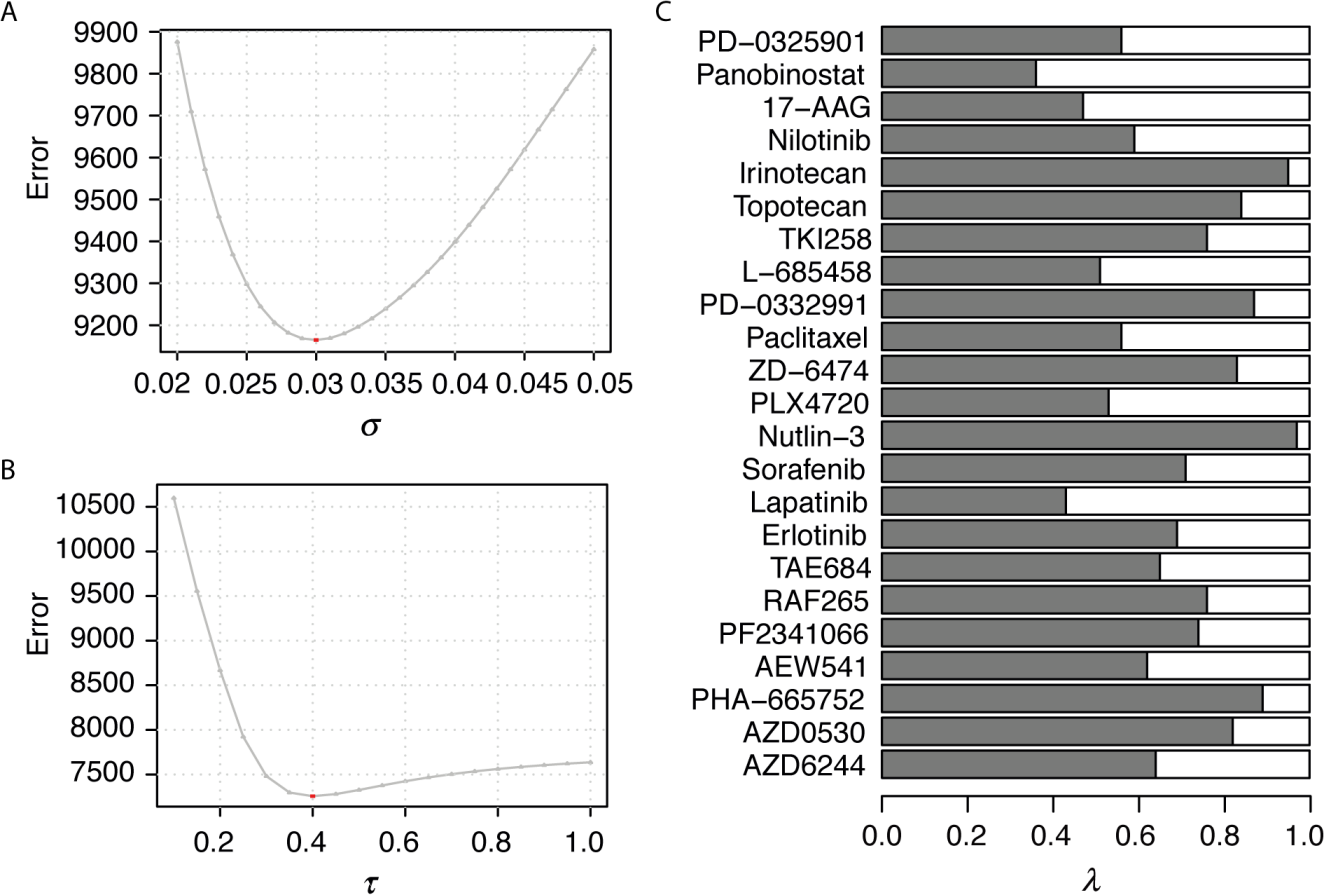
Parameter optimization for the dual-layer integrated cell line-drug network model. Using the CCLE study as an example, two bandwidth parameters, *σ* for the CSN (A) and *τ* for the DSN (B), were optimized separately. The optimized weighted parameter, λ, was determined for each of the 23 drugs in the CCLE study (C).

Our model contains only three parameters, which determine how to weigh the different cell lines *w* ^*C*^, how to weigh the different drugs *w* ^*D*^, and how to combine them λ, respectively. *w* ^*C*^ can in turn to be written as $w^{C}(C,C_{i})=e^{-\frac{[1-{\rho^{C}(C,C_{i})]}^{2}}{2\sigma^{2}}}$, where σ determines the decay rate when cell expression correlation decrease. Within the σ range of [0, 1] at 0.001 increments, we examined the top layer CSN and identified the optimal σ = 0.030 that minimizes sum of squares in prediction errors of all drugs in the CCLE dataset (Fig 3A). *w* ^*D*^ can also in turn be written as $w^{D}(D,D_{j})=e^{-\frac{[1-{\rho^{D}(D,D_{j})]}^{2}}{2\tau^{2}}},$ where τ determines the decay rate when drug similarity correlations decrease. Within the τ range of [0, 1] at 0.01 increments, we examined the bottom layer DSN and identified the optimal τ = 0.40 that minimizes sum of squares in prediction errors among all cell lines in the CCLE dataset (Fig 3B). Once σ (hence *w* ^*c*^) and τ (hence *w* ^*D*^) were determined, we calculated λ for each drug (Fig 3C), the parameter weighting the relative contributions of CSN and DSN, to minimize the sum of the squared errors. Most of the drugs have λ larger than 0.5 (Fig 3C), suggesting that DSN is more informative for predicting the drug response than CSN. The final prediction model for each drug contained the three optimized parameters.

### Cross-validation on CCLE and CGP drug response datasets

**Fig 4:**
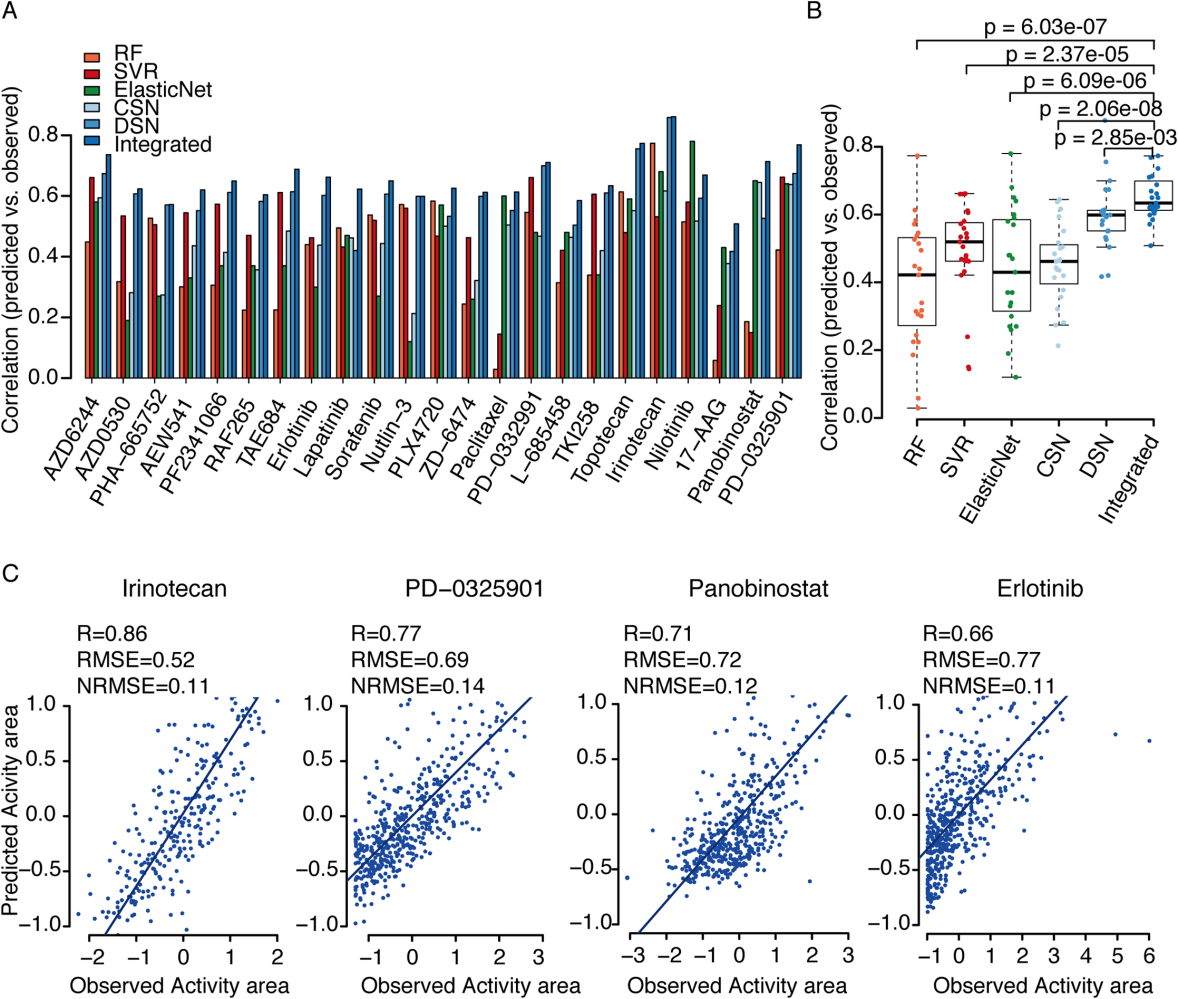
Prediction performance of the dual-layer integrated cell line-drug network model using the CCLE dataset. (A) Bar graph showing the prediction performance of six different models for 23 drugs tested in the CCLE study, which were quantified based on Pearson correlations between the predicted and observed activity areas. RF: prediction result by random forest using combined features of genomic and chemical data; SVR: prediction result by support vector regression using combined features of genomic and chemical data; CSN: prediction result using only the cell line similarity network; DSN: prediction result using only drug similarity network; ElasticNet: result from the CCLE study using the elastic net model; Integrated: prediction result using the dual-layer integrated cell line-drug network model that integrates CSN and DSN; (B) Pearson correlation distributions of the RF, SVR, CSN, DSN, ElasticNet and Integrated models, where t-tests were used to measure differences between two different groups. (C) Scatter plots of observed and predicted drug responses (activity area) for four drugs in CCLE using the dual-layer integrated cell line-drug network model.

In order to test the robustness of the estimated parameters in our dual-layer integrated cell line-drug network model, we conducted leave-one-out cross-validation by singling out each drug-cell line pair as the test dataset. Performance was measured by the Pearson correlation between predicted and observed drug responses. We first applied our method to the CCLE dataset, and compared the results to those from elastic net regression as reported in the original study (Fig 4A). Consistent with the above finding that λ is greater than 0.5 for most drugs, predictions based on DSN alone were better than those based on CSN alone for most drugs. In addition, the dual-layer model integrating DSN and CSN gave superior performance to either CSN alone or DSN alone, indicating that more information (genomic features of cell lines and chemical features of drugs) are helpful in drug response prediction. Three drugs, PD-0332991, Irinotecan, and Nutlin-3, showed similar performance between the dual-layer integrated cell line-drug network model and DSN alone, since their λ parameters are close to 1. More importantly, our predictions using the dual-layer network model were significantly better than the previously published predictions using the elastic net model for most drugs, except Nilotinib. Notably, the drugs AZD530 and Nutlin-3 had poor prediction correlations using the elastic net model (< 0.2), although both were well predicted by our dual-layer integrated cell line-drug network model (> 0.6). The overall prediction performance of the dual-layer integrated cell line-drug network model across all the drugs was significantly higher than that of the elastic net model for the CCLE dataset (Fig 4B). We believe that the improved performance of our model is partially due to the incorporation of drug chemical features, which are not used in the previous elastic net model. The scatter plots of observed versus predicted responses for 4 example drugs (Fig 4C) indicate that the good correlations did not arise from a small number of outliers (the remaining 19 drugs are listed in S2 Fig).

**Fig 5:**
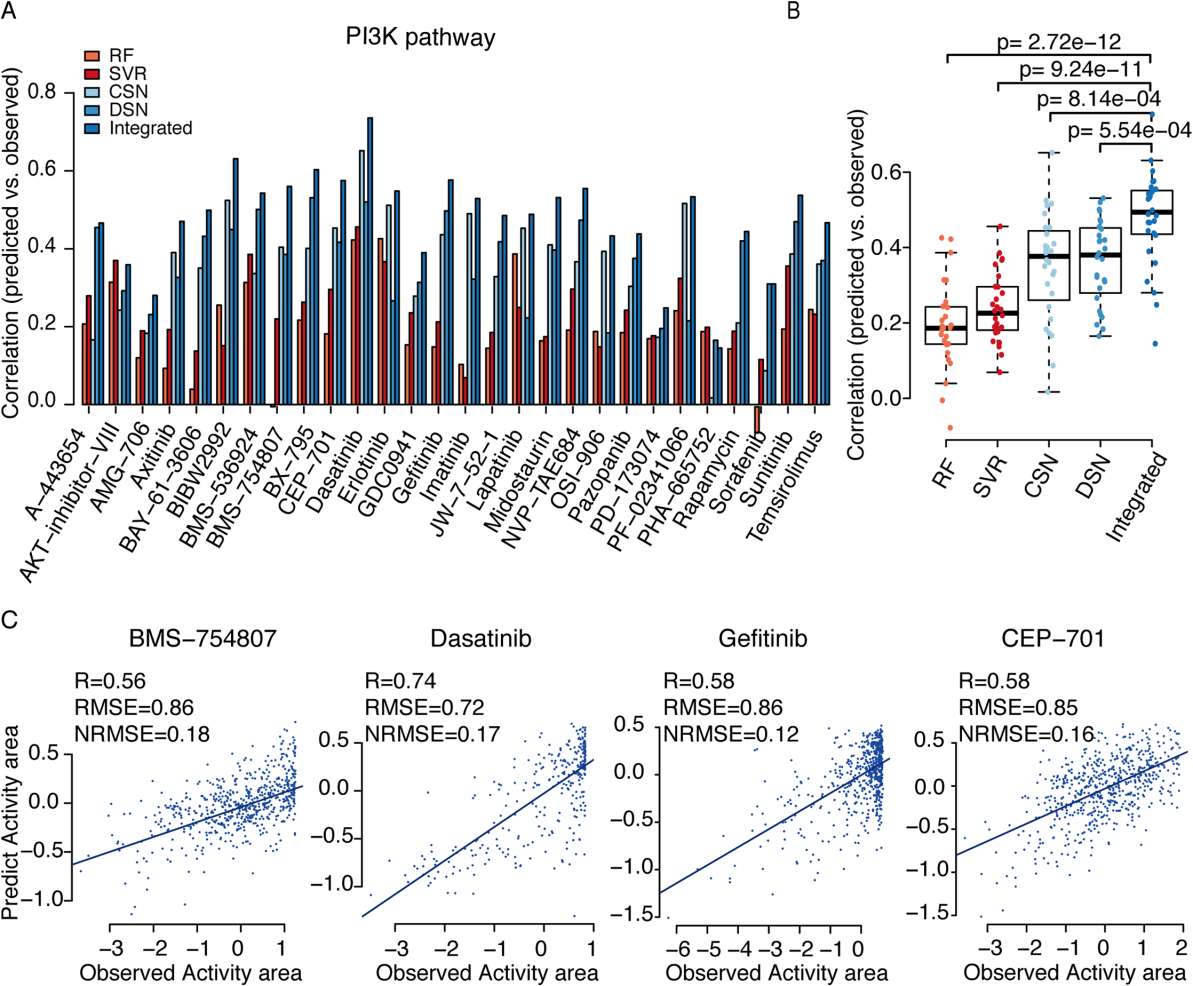
Prediction performance of the dual-layer network model for drugs targeting the PI3K pathway in CGP. **(**A) Bar graph showing the prediction performance of five models using experimental data from the CGP study, which was quantified using the Pearson correlation between predicted and observed activity areas. RF: prediction result by random forest using combined features of genomic and chemical data; SVR: prediction result by support vector regression using combined features of genomic and chemical data; CSN: prediction results using only the cell line similarity network; DSN: prediction results using only the drug similarity network; Integrated: prediction results using the dual-layer integrated cell line-drug network model. (B) Pearson correlation distribution of the five different models using t-tests to determine differences between two groups. (C) Correlations between observed and predicted activity areas using the dual-layer integrated cell line-drug network model.

We also conducted leave-one-out cross-validation to evaluate the performance of the dual-layer integrated cell line-drug network model in the CGP study with activity area as drug response measurement. For drugs targeting genes in the PI3K (Fig 5A and 5B) and ERK (S3 Fig) pathways, the dual-layer integrated cell line-drug network model gave consistently better performance than CSN or DSN alone. In addition, for around half of these drugs, the Pearson correlations between the observed and predicted responses from the dual-layer integrated cell line-drug network model were higher than 0.5 (Fig 5A and S3A Fig). Scatter plots of 4 example drugs also indicate that the good correlations are fairly reasonable with relatively small numbers of outliers (Fig 5C and S3C Fig), results of the rest drugs in these two pathways are shown in (S4 Fig). Similar conclusion can be drawn when using IC50 as drug response measurement (S5 Fig).

We next compared our network models with a machine-learning approach which also predicts drug response based on genomic feature of cell lines and chemical features of drugs [17]. Basically, it treats drug response prediction as a machine-learning problem where each possible drug-cell line pair is represented through integrating genomic features of the cell line and chemical structural features of the drug. Neural network (NN) and random forest (RF) are then used to build a prediction model based on training data. However, NN gave very poor performance despite our numerous attempts to set different parameters, so we test RF and another user-friendly machine-learning tool, support vector regression (SVR). For CCLE and CGP datasets, the overall correlation between observed activity areas and predicted from SVR and RF (S6 Fig) among all drugs and all cell lines is high and almost the same as [17]. However, since the baseline of different drugs have very different activity area ranges (S7 Fig), this overall correlation doesn’t indicate how a particular drug will behave on different cells. For each individual drug, the correlations are only around 0.20 (RF) and 0.23 (SVR) in CGP, and 0.49 (RF) and 0.38 (SVR) in CCLE, which are often worse than our single layer model as well as the elastic net model, and much less than our integrated model (Figs 4A, 4B, 5A and 5B). In addition, the method in their model treats every possible drug-cell line pair as an individual case, so it is computationally expensive for larger number of drugs and cell lines, especially for CGP. But our model only relies on correlations between cell lines or drugs, thus is computationally efficient.

However, as pointed by many researches, leave-one-out cross-validation in regression will possibly overestimate the accuracy of predictions on out-of-sample observations due to over fitting. To assess the potential bias in above leave-one-out cross-validation, we randomly shuffled response values of all cell lines to each drug, then repeated our predictions with the same parameter settings [25]. As is shown in (S8 Fig), for all drugs tested in CCLE and CGP, we observed only very weak correlations between real and predicted values (absolute correlations are less than 0.08 for more than 95% drugs), indicated that our model is not biased by the cross-validation procedure.

### Missing data estimation in the CGP drug response matrix

**Fig 6:**
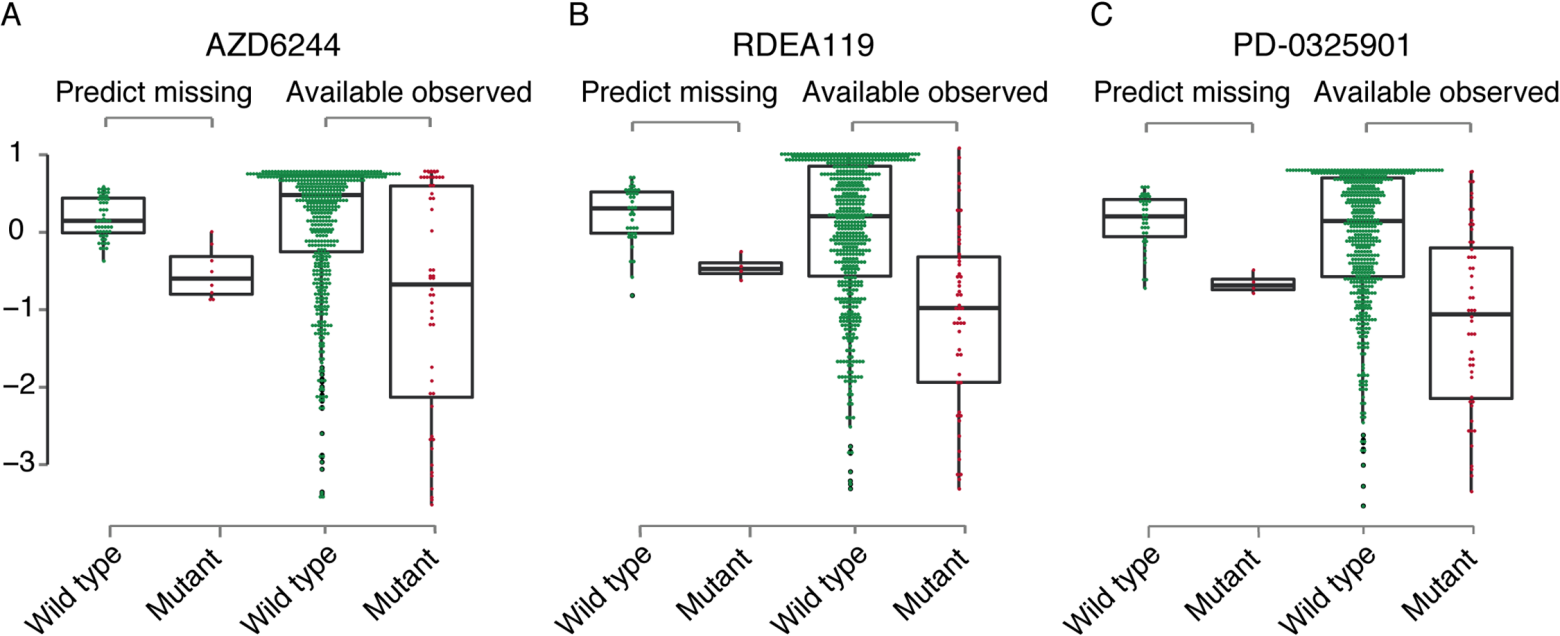
Comparison of predicted and observed activity areas using the dual-layer integrated cell line-drug network model for BRAF mutant and wild-type cell lines for which experimental activity areas was missing from the CGP dataset for three MEK1/2-inhibitors, including (A) AZD6244, (B) RDEA119 and (C) PD-0325901.

Although the CGP study used a total of 707 cell lines and 139 drugs, only 653 cell lines had expression profiles and only 124 drugs had chemical information available. Out of the possible 653 × 124 cell line-drug combinations, only 76% have corresponding drug response data. With the cell similarity and drug similarity data, we could use our dual-layer integrated cell line-drug network model to predict the missing activity areas (Fig 6) and IC50 (S9 Fig), with a particular focus on three MEK inhibitors AZD6244, RDEA119, and PD-0325901, where a large number of response values were missing. When grouping the cell lines based on their BRAF mutation profiles, we found that the BRAF-mutated cell lines were significantly more sensitive to MEK inhibitors (Fig 6). These predictions were consistent with those in cell lines where response data were available, and were in agreement with previously published studies. The above findings suggest that our dual-layer integrated cell line-drug network model can be used to optimize the design of cell line screens with new drugs by combining *in silico* predicted response values from existing screen results and the structure of the new drugs.

## Discussion

In this study, we developed a dual-layer integrated cell line-drug network model to predict the response of cancer cell lines to drug treatments for experimental activity areas in the CCLE and IC50 values in the CGP study. The novelty of our method lies in the incorporation of two distinct sub-networks for predicting drug responses: 1) a cell similarity network (CSN), based on similarities in gene expression profiles between cell lines, and 2) a drug similarity network (DSN), based on similarities in chemical structural features between drugs. This method applies a weighted algorithm to model the predictor. Using the data from the CCLE and CGP studies as benchmarks, leave-one-out cross-validation showed that our dual-layer integrated cell line-drug network model consistently outperformed the elastic net model, suggesting that our model effectively captures the interplay in the cell line-drug network. Our model also reasonably assigned much lower activity areas for BRAF mutant cell lines than for BRAF wild-type cell lines for three different MEK1/2-inhibitors, which is in good agreement with current experimental reports. Our network model can be applied to predict the response of a new cell line to existing already tested drugs or to predict the response of an existing cell line to new drugs, thus potentially saving the cost in a drug-cell line screening. Compared to the existing elastic net regression, our dual-network model is based on the entire dataset rather than a single drug, so interactions between different drugs are considered. Furthermore, our model only needs correlations between cell lines or drugs as input, thus is not seriously affected by the huge dimensionality of genome-wide gene expression and copy number variation features.

Despite these encouraging results, our model suffers from the following limitations, which we hope to address in the future. First, construction of the CSN relied solely on gene expression profile data, and future work to integrate somatic mutation information, epigenetic status [26,27], and pathways could potentially improve the performance of our network models. Furthermore, some other dynamic and direct features, such as time-course gene expression [28] or protein signaling [29], might better illustrate a cell line and potentially give a more reliable CSN. Second, construction of the DSN used the 1-D and 2-D structural features of each drug, which might provide too many features for small molecule drugs, and yet ignored the 3-D structure features which may be important for certain drugs. Third, our dual-network model is a local weighted model based on responses of existing cell lines to the query drug or (and) the query cell line to other similar drugs by chemical structure. So it does not work without the above two types of information, such as the response prediction for a new drug to a new cell line, which is more useful in real application cases. With increasing data on cell-line drug responses becoming available over time, and extended network models to address the above limitations, we hope this network-based approach will have much better predictive power and potentially be used in drug combination exploration [30]. Finally, our local weighted model could possibly shrink the range of predicted drug responses and thus lead to a quite large RMSE. For example, if response of a drug *d* and cell line *c* is the largest among all training data, the linear combination of response values from their neighborhood will definitely be smaller than the truth. However, in practical clinical settings, one may care more about the relative order rather than the absolute values of drug response data due to the batch effect of different experiments. So most work in drug response prediction used correlation between true and predicted values as a measure of effectiveness [13,17], which might be a better measure than RMSE. In fact, even the published original data from CCLE and CGP have different magnitudes in IC50 for their common drugs (S7 Fig).

## Materials and Methods

### Data resources

The cancer genomic and drug response data used in this work are available from the Cancer Cell Line Encyclopedia (CCLE) [13] and the Cancer Genome Project (CGP) [14]. The CCLE dataset consists of large-scale genomic data, including gene expression profiles, mutation status, and copy number variation for 1,036 human cancer cell lines, and eight-point dose-response curves for 24 chemical compounds across 504 cell lines. Gene expression profiles and drug sensitivity data (measured by area under dose-response curves) were downloaded from the CCLE website (http://www.broadinstitute.org/ccle). Both drug sensitivity measurements and gene expression profile data are available for 491 common cancer cell lines. The CGP dataset provided raw gene expression profiles for 789 cell lines, which we downloaded from AffyExpress under accession number E-MTAB-783. We also obtained drug sensitivity measurements (activity area) and cell line annotation data from the CGP website (http://www.cancerrxgene.org/downloads), which included data for 139 drugs and 707 cancer cell lines. The CGP study provided both drug sensitivity measurements and gene expression profile data for 653 common cancer cell lines.

We downloaded the chemical structural information for each drug from PubChem, a database of chemical molecules and their activities in different biological assays. It contains validated chemical depiction information for 19 million unique compounds contributed from over 70 depositing organizations. We downloaded raw chemical property profiles (SDF files) for 23 drugs in the CCLE study and 124 drugs in the CGP study from the PubChem website. The 1-D and 2-D chemical structural features of each drug were retrieved using the PaDEL software program (v2.11, downloaded from the project website http://padel.nus.edu.sg/software/padeldescriptor/) with default settings. In detail, the 1D descriptors consist of compositional or constitutional molecular properties, e.g., atom count, bond count, and molecular weight. The 2D descriptors include different quantitative properties of the topology such as Kappa shape indices [31], Randic [32] and Wiener indices [33] (S1 Table).

### Construction of the drug-cell line integrated network

Our integrated network for predicting drug responses consists of three parts: 1) a cell line similarity network (CSN), which connects all cell lines based on their Pearson correlation coefficients of gene expression profiles; 2) a drug similarity network (DSN), where associations between two drugs are measured by the Pearson correlation between their respective 1-D and 2-D structural properties; and 3) a drug-cell line response network, which connects the above two networks using experimentally determined drug response values (measured as activity area and IC50 in both CCLE and CGP studies). The CSNs generated using CCLE or CGP data are both complete graphs of all cell lines, where interactions between two cell lines are measured by the Pearson correlation coefficients of their respective gene expression profiles. Similarly, the DSNs are also complete graphs of all available drugs, connected with their pairwise correlation between their respective 1-D and 2-D chemical structural features. The intermediate layer between the CSN and the DSN, referred to as the drug-cell line response network, is a bipartite graph of all cell lines and drugs, labeled with the response values (corresponding to activity area and IC50 values). Note that the intermediated drug response network is not a complete bipartite graph due to some missing values in two experiments, particularly in the CGP dataset.

### Predicting a drug response for a new cell line based on the cell line similarity network

In order to predict the response of a new cell line *C* to a known drug *D*, similar to the LWR (Locally weighted linear regression), we take advantage of data for all neighboring cell lines to cell line *C* in the cell line similarity network (CSN). For drug *D* that is being investigated, neighboring cell lines are excluded from the analysis if drug sensitivity data is not available for them. Based on the hypothesis that cell lines with similar gene expression profiles will respond similarly to the same drug, we propose a linear weighted model to approximate the sensitivity of cell line *C* to drug *D* as follows:

$${\hat{Sens}}_{CSN}(D,C)=\frac{\sum_{C_{i\neq }C}Sens(D,C_{i})w^{C}(C,C_{i})}{\sum_{C_{i\neq }C}w^{C}(C,C_{i})}$$ (1)

where *Sens*(*D*,*C*_*i*_) is the sensitivity data of cell line *C*_*i*_ against drug *D*, *w* ^*C*^(*C*,*C*_*i*_) is the weight parameter, and *C*_*i*_ is the sample that is associated with cell line *C* in the CSN. According to our model assumption, the most closely related cell line in terms of its gene expression profile will contribute much more to the prediction of *Sens*(*D*,*C*) compared to other cell lines. Therefore, the weight parameter should be an increase function with their correlation to *C*. A fairly standard choice for the weights is:

$$w^{C}(C,C_{i})=e^{-\frac{[1-{\rho^{C}(C,C_{i})]}^{2}}{2\sigma^{2}}}$$ (2)

in which *ρ* ^*C*^(*C*,*C*_*i*_) is the gene expression correlation between cell line *C* and *C*_*i*_, σ is the bandwidth parameter controlling how quickly the weight of a training example falls off with distance of its *C*_*i*_ from the query point *C*. Here, if *ρ* ^*C*^(*C*,*C*_*i*_) is close to 1, *w* ^*C*^(*C*,*C*_*i*_) will also be close to 1, implying that this cell line has a high impact in the evaluation of *Sens*(*D*,*C*). On the contrary, if *ρ* ^*C*^(*C*,*C*_*i*_) is small (e.g., close to 0), *w* ^*C*^(*C*,*C*_*i*_) will be relatively small too. In this case, the corresponding cell line will have a weak contribution to the determination of the unknown cell line.

### Predicting a cell line response to a new drug based on the drug similarity network

In the previous section, we developed a model to predict the response of an unknown cell line to an existing drug based on the training data. However, in some cases, we may need to predict the response of a known cell line to a new drug. Based on a model similar to that used in the previous section, we propose a linear weighted model for predicting the sensitivity of known cell line *C* to a new drug *D* based on the known sensitivities of cell line *C* to all neighboring drugs to drug *D*. We developed a similar linear weighted model to predict the response of a cell line to a new drug based on its neighboring drugs in the drug similarity network (DSN) as follows:

$${\hat{Sens}}_{DSN}(D,C)=\frac{\sum_{D_{j}\neq D}Sens(D_{j},C)w^{D}(D,D_{j})}{\sum_{D_{j}\neq D}w^{D}(D,D_{j})}$$ (3)

where the weight *w* ^*D*^(*D*_*j*_,*C*) is determined by the correlation between the chemical structural feature of drug *D* and drug *D*_*j*_ in the drug similarity network:

$$w^{D}(D,D_{j})=e^{-\frac{[1-{\rho^{D}(D,D_{j})]}^{2}}{2\tau^{2}}}$$ (4)

where *τ* is the bandwidth parameter.

### Drug response prediction based on the dual-layer integrated cell line-drug network model

In the above two sections, we developed single-layer predictive models for determining the response of a cell line to a drug based on the cell line similarity network (CSN) and the drug similarity network (DSN), depending on whether drug and cell line information are available. However, the results from either of these single-layer models may be not satisfactory when only one type of information (either cell line similarity or drug similarity) is considered. To make full use of the integrated network, we propose a linear weighted model to combine ${\hat{Sens}}_{CSN}(D,C)$ and ${\hat{Sens}}_{DAN}(D,C)$ as follows:

$$\hat{Sens}(D,C)=\lambda ∙{\hat{Sens}}_{DSN}(D,C)+(1-\lambda )∙{\hat{Sens}}_{CSN}(D,C)$$ (5)

where *λ* is the combination weight, which can be optimized through leave-one-out cross-validation. The integrated model will be dominated by DSN if *λ* is close to 1, and it will be dominated by CSN if *λ* is close to 0. Two individual models are complementary when 0 < *λ* < 1.

### Leave-one-out cross-validation and evaluation criteria

We used the leave-one-out cross-validation method to determine the parameters and validate our predictor. In detail, each possible drug-cell line pair is singled out as the test dataset to measure the consistency between predicted and observed drug response with the model trained from the remaining data. There are three free parameters to be determined in our model, i.e., σ and τ which measure the decay rate when correlations of cell line expression or drug descriptors decrease, and λ which measures the relative contribution of each single layer. In order to ensure a unified model, two decay parameters σ and τ are fixed for all drugs and cell lines in CSN and DSN respectively, but λ is optimized by each individual drug, allowing different relative contributions of two lays for different drugs.

Three parameters are optimized in the following order to make sure drug response can be predicted by each individual layer and the whole integrated network. For the cell line similarity network, the decay parameter σ is optimized by minimizing sum of squared prediction errors for all possible drug-cell line combinations using Formula (1) as prediction model. In detail, the overall error function (or cost function) is defined as:

$$J(\sigma )=\sum_{D,C}{\left(Sens(D,C)-{\hat{Sens}}_{CSN}(D,C)\right)}^{2}$$ (6)

where *Sens*(*D*,*C*) is the observed sensitivity value of cell line *C* to drug *D*, and ${\hat{Sens}}_{CSN}(D,C)$ is the predicted one through cell line similarity models using all other drug-cell line interactions as the training set. The best parameter was obtained by minimizing the error function as follows:

$$\hat{\sigma }={\mathrm{argmin}}_{\sigma }\;J(\sigma ).$$

Similarly, decay parameter *τ* for the bottom drug similarity network was determined by

$$\hat{\tau }={\mathrm{argmin}}_{\tau }\;\sum_{D,C}{\left(Sens(D,C)-{\hat{Sens}}_{DSN}(D,C)\right)}^{2}$$ (7)

Finally, the best λ for each drug is obtained through minimizing the sum of squared errors after combining predictions from both CSN and DSN, i.e.,

$$\hat{\lambda }={\mathrm{argmin}}_{\lambda }\;\sum_{C}{\left(Sens(D,C)-\hat{Sens}(D,C)\right)}^{2}.$$ (8)

After selecting the parameters, the prediction performance of our model was evaluated using Pearson correlation coefficient, root mean squared error (RMSE) and normalized root mean squared error (NRMSE) between predicted and observed drug responses for each drug. RMSE is the square root of the mean squared error,

$$RMSE(D)=\sqrt{\frac{\sum_{c}{\left(Sens(D,C)-\hat{Sens}(D,C)\right)}^{2}}{n}},$$ (9)

and NRMSE is got by dividing the range of drug responses to facilitate the comparison between different drugs or predictive models,

$$NRMSE(D)=\frac{RMSE(D)}{\max_{C}Sens(D,C)-\min_{C}Sens(D,C)}.$$ (10)

A higher Pearson correlation coefficient and lower RMSE/NRMSE indicate a better prediction performance of a method for a given drug.

## Supporting Information

S1 Fig Model assumption by IC50 as drug sensitivity measurement. (A, B) Box plots showing cell lines with similar gene expression profiles responding similarly to the same drugs. The X-axis indicates the Pearson correlation coefficients between all possible cell line pairs based on expression profiles. The Y-axis shows the correlations of their drug response vectors as measured by IC50 in CCLE (A) and CGP (B). (C, D) Box plots showing that drugs with similar 1-D and 2-D structural features based on PaDEL exhibiting similar effects on cell lines in the CCLE (C) and CGP (D) datasets. The X-axis represents the drug similarity categories, and the Y-axis shows the correlations of drug responses across all cell lines. Statistical differences between two groups were measured by the t-test.
(TIF)

S2 Fig Scatter plots of observed and predicted Activity areas for drugs in the CCLE dataset. (TIF)

S3 Fig Prediction performance of the dual-layer network model in CGP for drugs targeting the ERK signaling pathway by drug-response activity area. (A) Bar graph showing the prediction performance of three models using experimental data from the CGP study, quantified using the Pearson correlation between the predicted and observed activity areas. (B) Pearson correlation distribution of the three different models using t-tests to determine differences between two groups. (C) Correlations between observed and predicted activity areas.
(TIF)

S4 Fig Scatter plots of observed and predicted Activity areas for drugs targeting the PI3K and ERK Signaling pathway in the CGP dataset. (TIF)

S5 Fig Prediction performance of the dual-layer network model for the CGP dataset by IC50. **(**A) Bar graph showing the prediction performance of three models for drugs targeting the PI3K pathway using experimental data from the CGP study, which was quantified using the Pearson correlation between predicted and observed IC50 values. CSN: prediction results using only the cell line similarity network; DSN: prediction results using only the drug similarity network; Integrated: prediction results using the dual-layer integrated cell line-drug network model. (B) Pearson correlation distribution of the three different models using t-tests to determine differences between two groups. (C) Correlations between observed and predicted IC50 values for four drugs targeting the PI3K pathway using the dual-layer integrated cell line-drug network model. (D) Bar graph showing the prediction performance of three models for drugs targeting the ERK signaling pathway using experimental data from the CGP study, quantified using the Pearson correlation between the predicted and observed IC50 values. (E) Pearson correlation distribution of the three different models using t-tests to determine differences between two groups. (F) Correlations between observed and predicted IC50 values for four drugs targeting the ERK Signaling pathway.
(TIF)

S6 Fig Correlation between predicted to observed activity areas for all drug-cell line pairs in (A) CCLE and (B) CGP. Support vector regression and random forest are used as predictors.
(TIF)

S7 Fig Drug sensitivity distribution of common drugs between CCLE and CGP. (TIF)

S8 Fig Correlations of predicted and observed drug responses before and after randomly shuffling the drug response values for (A) CCLE and (B) CGP. (TIF)

S9 Fig Comparison of predicted and observed IC50 values using the dual-layer integrated cell line-drug network model for BRAF mutant and wild-type cell lines for which experimental IC50 data was missing from the CGP dataset for three MEK1/2-inhibitors, including AZD6244 (A), RDEA119 (B), and PD-0325901 (C). (TIF)

S1 Table 1D and 2D chemical descriptors of drugs extracted from PaDEL. (XLS)
