# Supplementary figures and images for: Predicting Anticancer Drug Responses Using a Dual-Layer Integrated Cell Line-Drug Network Model
Source: PLoS Comput Biol. 2015 Sep 29;11(9):e1004498. doi: 10.1371/journal.pcbi.1004498 (PMC4587957; doi:10.1371/journal.pcbi.1004498)

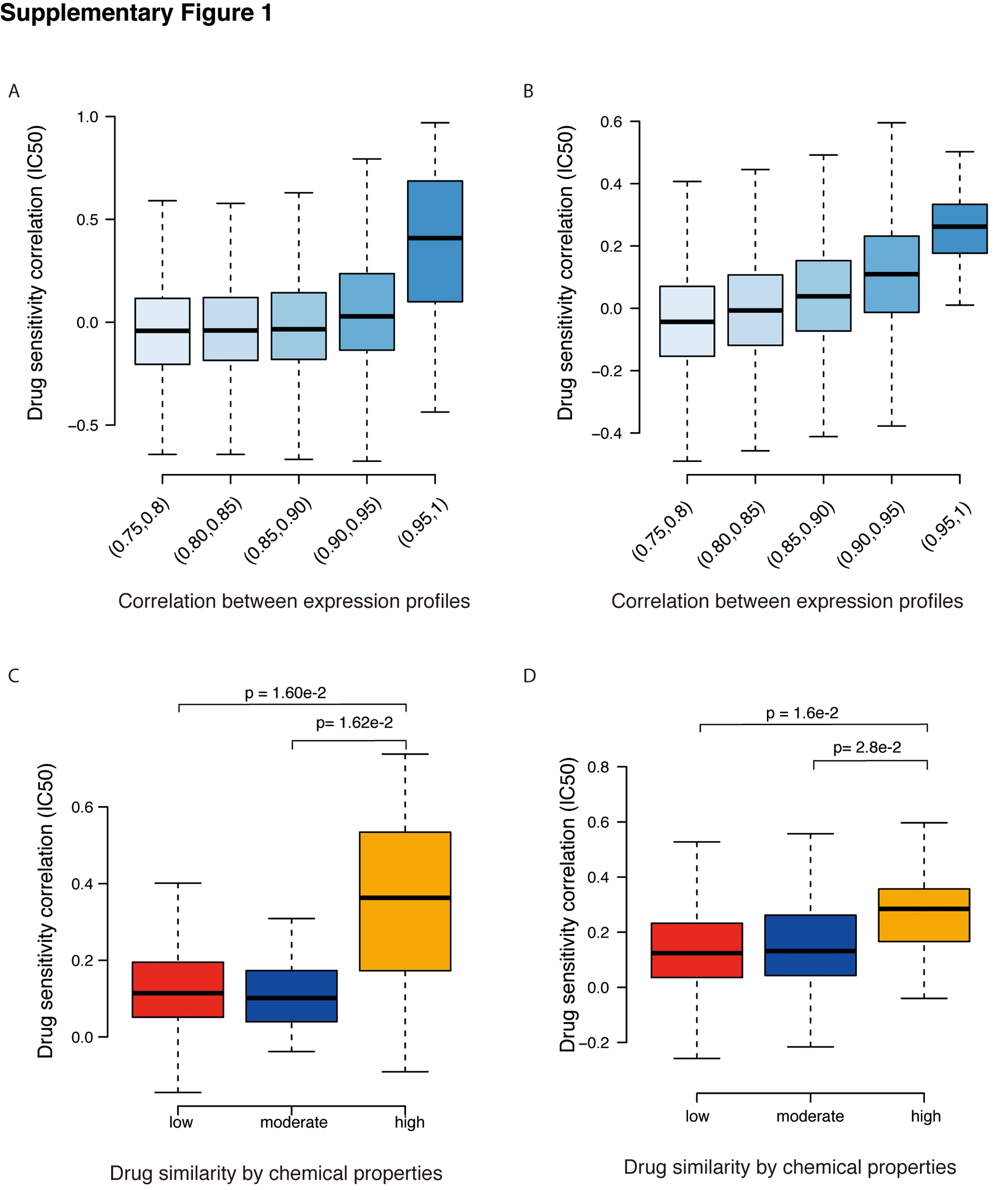

Supplement: S1 Fig — (A, B) Box plots showing cell lines with similar gene expression profiles responding similarly to the same drugs. The X-axis indicates the Pearson correlation coefficients between all possible cell line pairs based on expression profiles. The Y-axis shows the correlations of their drug response vectors as measured by IC50 in CCLE (A) and CGP (B). (C, D) Box plots showing that drugs with similar 1-D and 2-D structural features based on PaDEL exhibiting similar effects on cell lines in the CCLE (C) and CGP (D) datasets. The X-axis represents the drug similarity categories, and the Y-axis shows the correlations of drug responses across all cell lines. Statistical differences between two groups were measured by the t-test. (TIF) [file pcbi.1004498.s001.tif]

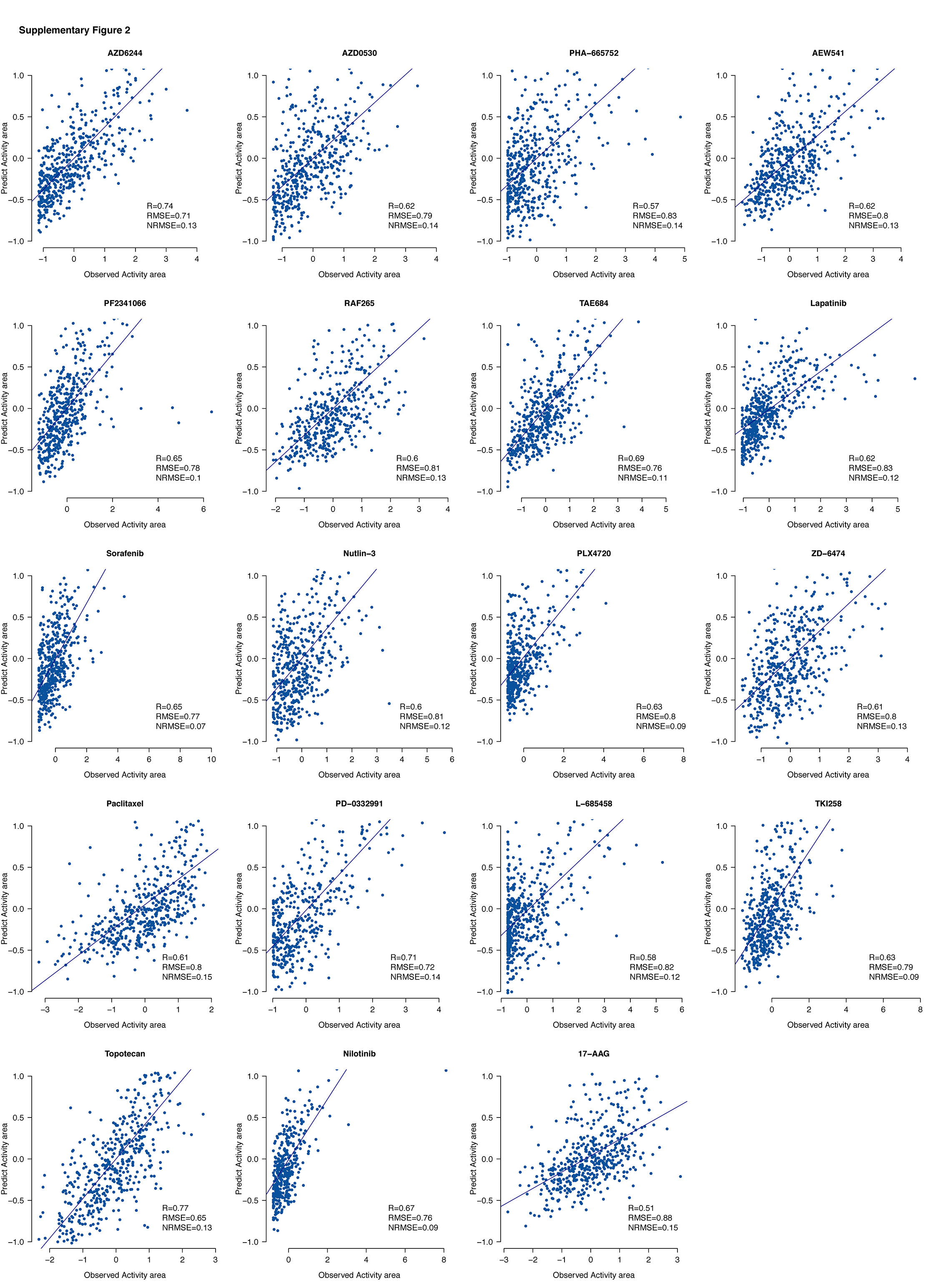

Supplement: S2 Fig — (TIF) [file pcbi.1004498.s002.tif]

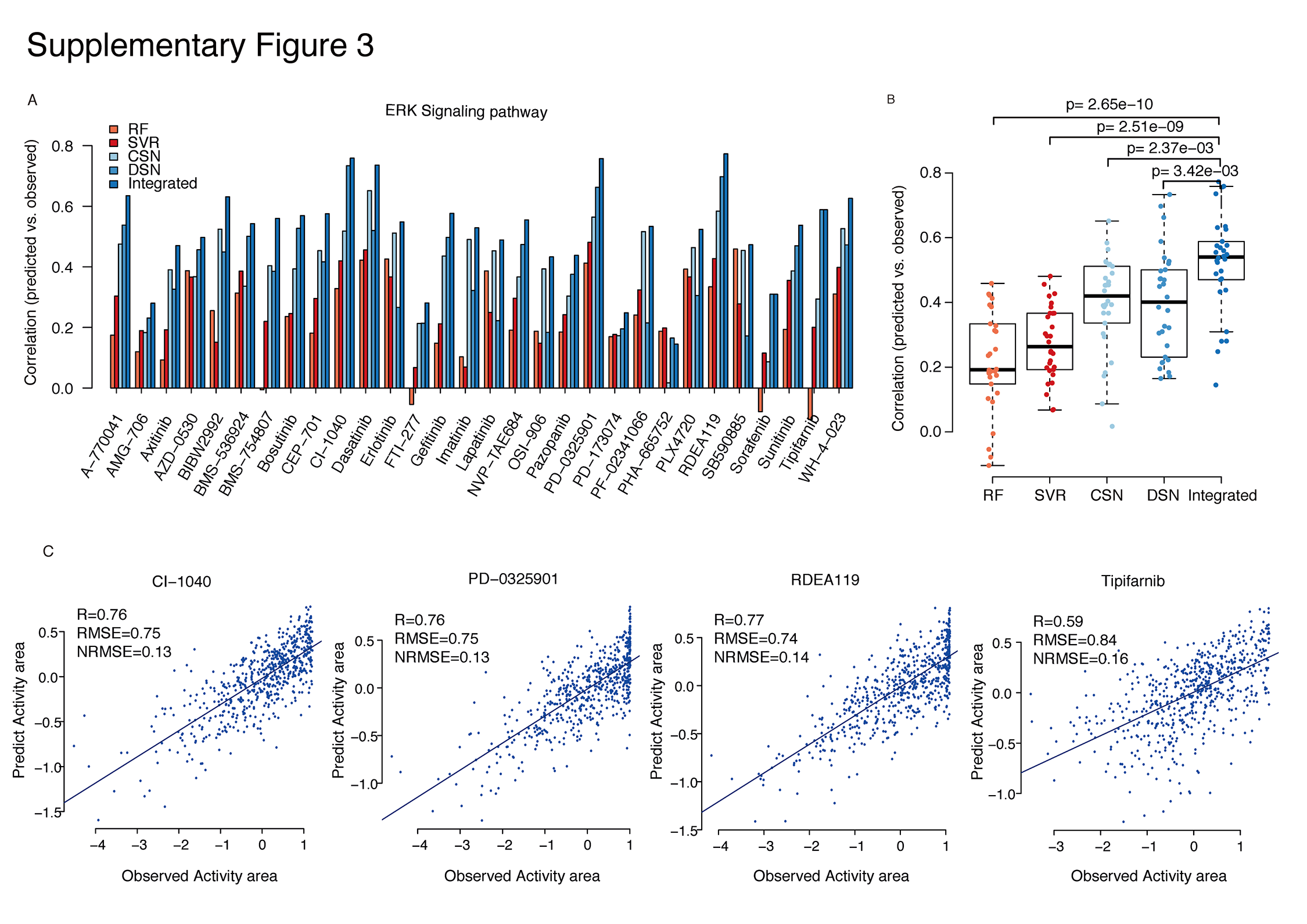

Supplement: S3 Fig — (A) Bar graph showing the prediction performance of three models using experimental data from the CGP study, quantified using the Pearson correlation between the predicted and observed activity areas. (B) Pearson correlation distribution of the three different models using t-tests to determine differences between two groups. (C) Correlations between observed and predicted activity areas. (TIF) [file pcbi.1004498.s003.tif]

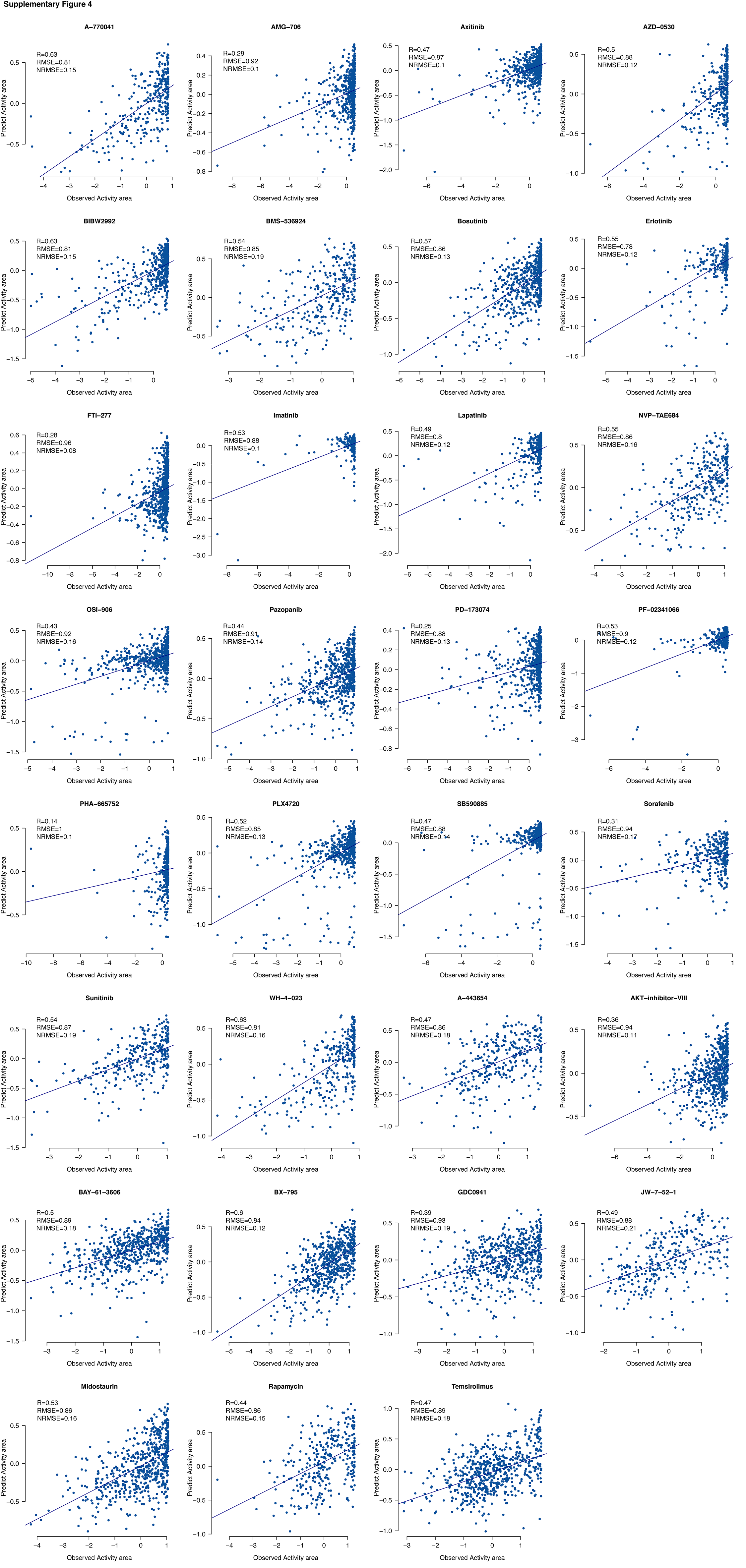

Supplement: S4 Fig — (TIF) [file pcbi.1004498.s004.tif]

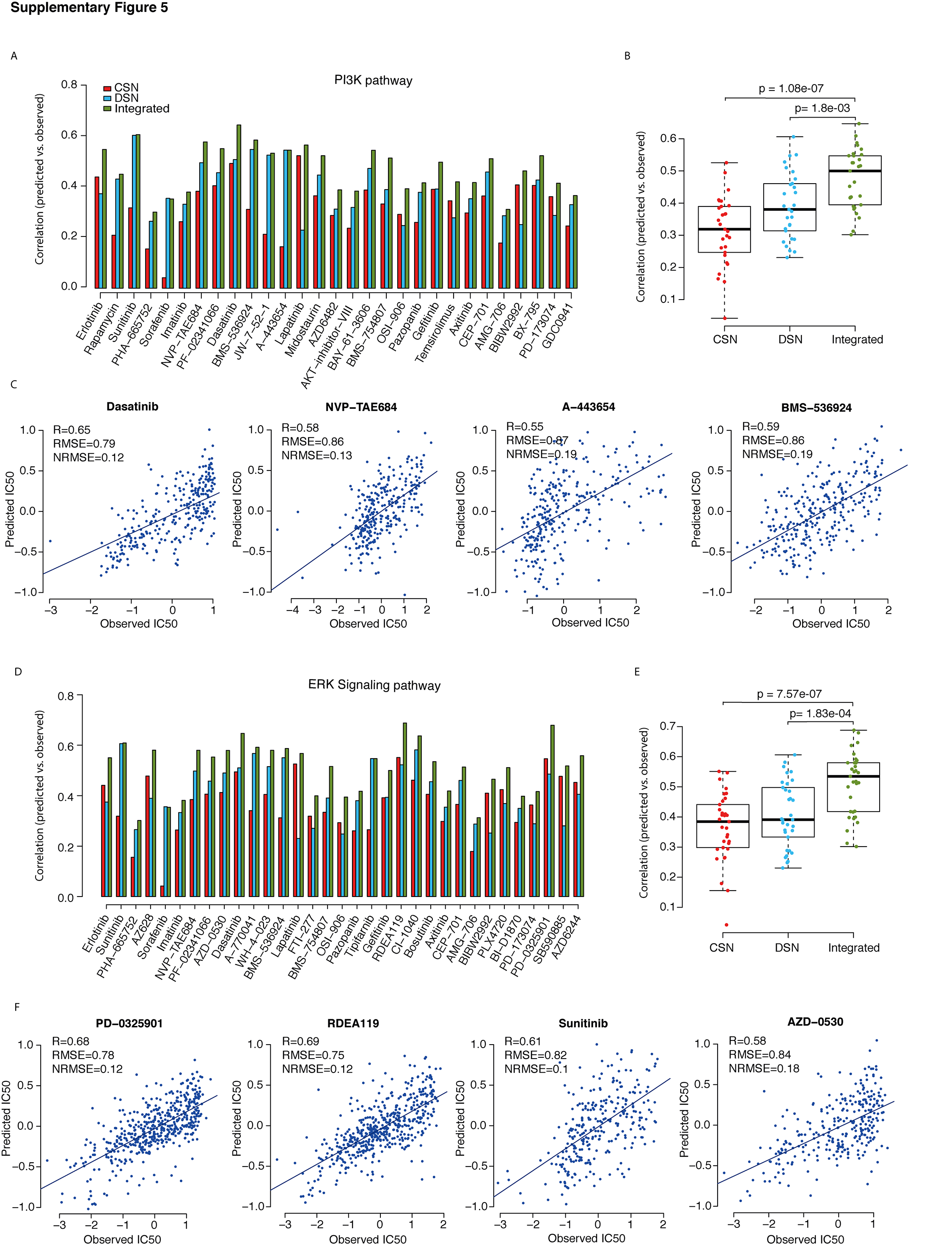

Supplement: S5 Fig — (A) Bar graph showing the prediction performance of three models for drugs targeting the PI3K pathway using experimental data from the CGP study, which was quantified using the Pearson correlation between predicted and observed IC50 values. CSN: prediction results using only the cell line similarity network; DSN: prediction results using only the drug similarity network; Integrated: prediction results using the dual-layer integrated cell line-drug network model. (B) Pearson correlation distribution of the three different models using t-tests to determine differences between two groups. (C) Correlations between observed and predicted IC50 values for four drugs targeting the PI3K pathway using the dual-layer integrated cell line-drug network model. (D) Bar graph showing the prediction performance of three models for drugs targeting the ERK signaling pathway using experimental data from the CGP study, quantified using the Pearson correlation between the predicted and observed IC50 values. (E) Pearson correlation distribution of the three different models using t-tests to determine differences between two groups. (F) Correlations between observed and predicted IC50 values for four drugs targeting the ERK Signaling pathway. (TIF) [file pcbi.1004498.s005.tif]

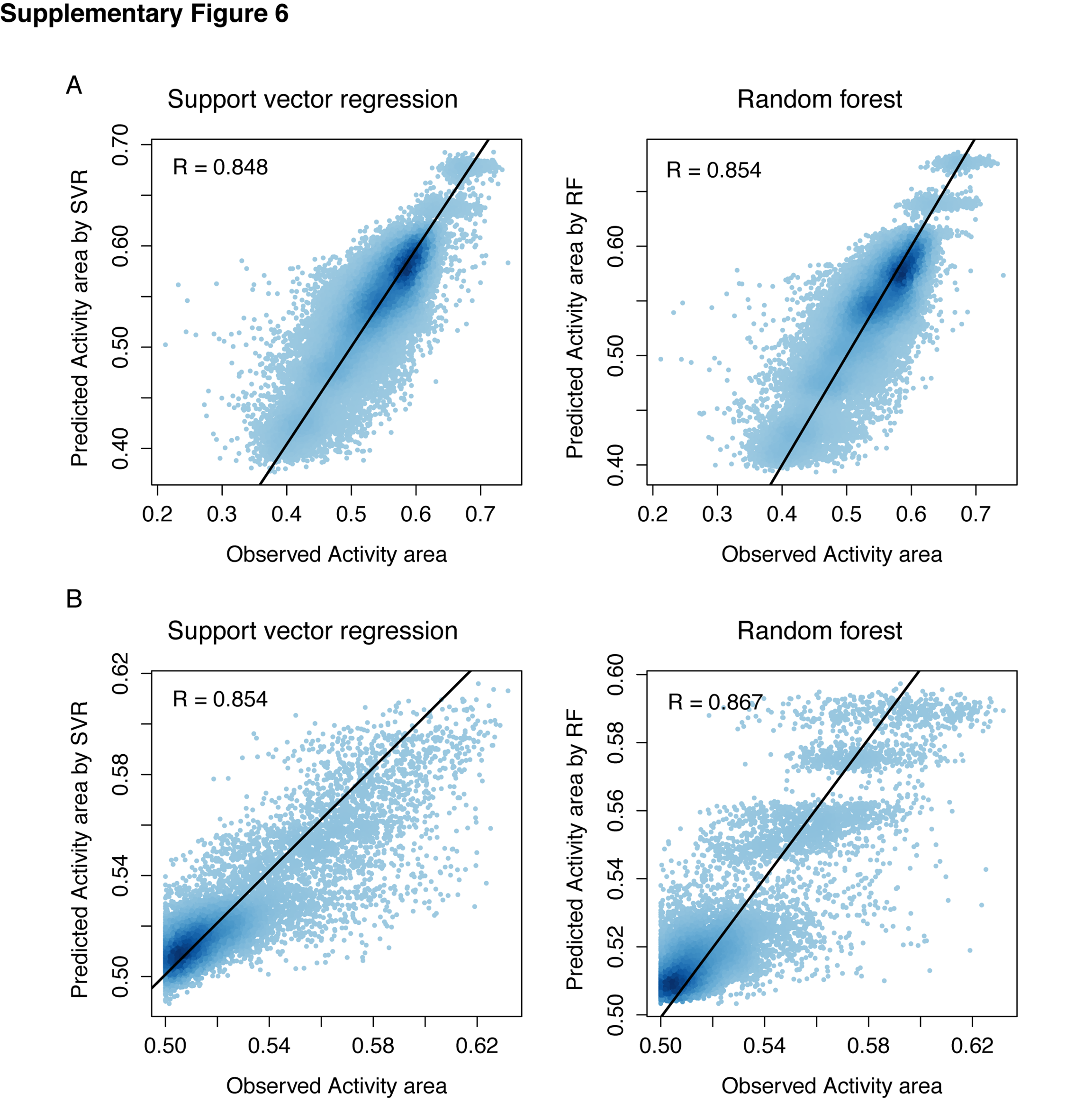

Supplement: S6 Fig — Support vector regression and random forest are used as predictors. (TIF) [file pcbi.1004498.s006.tif]

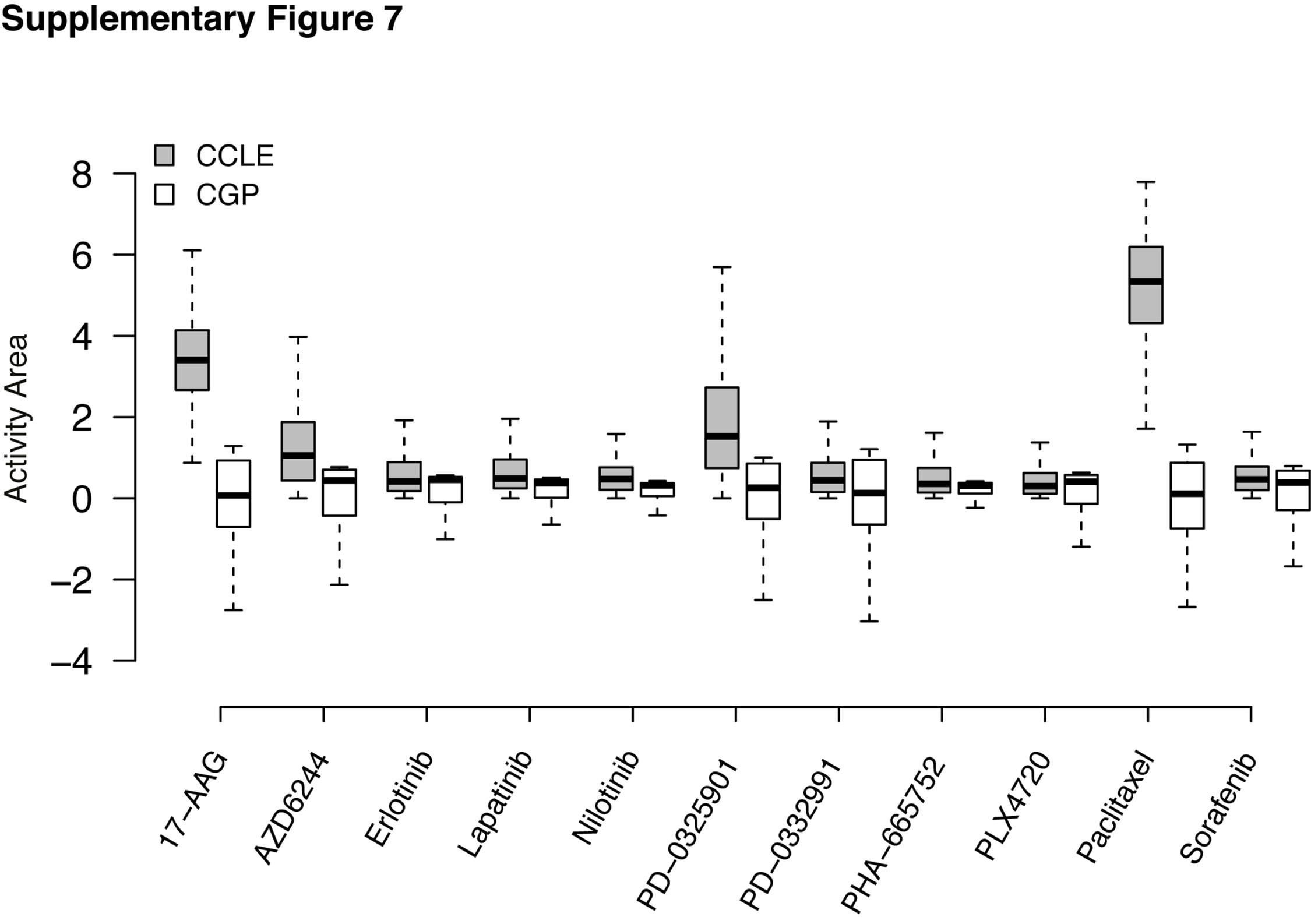

Supplement: S7 Fig — (TIF) [file pcbi.1004498.s007.tif]

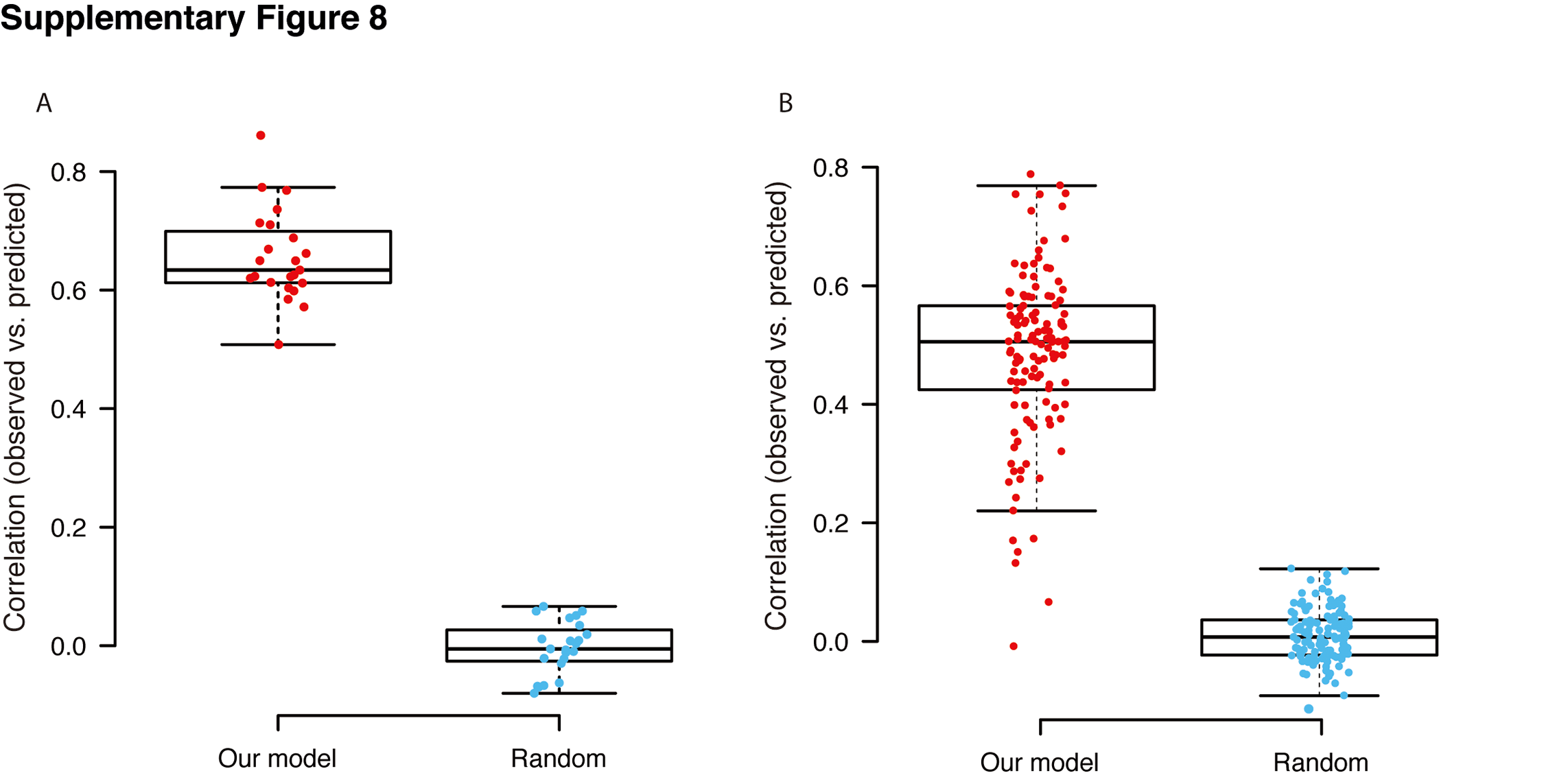

Supplement: S8 Fig — (TIF) [file pcbi.1004498.s008.tif]

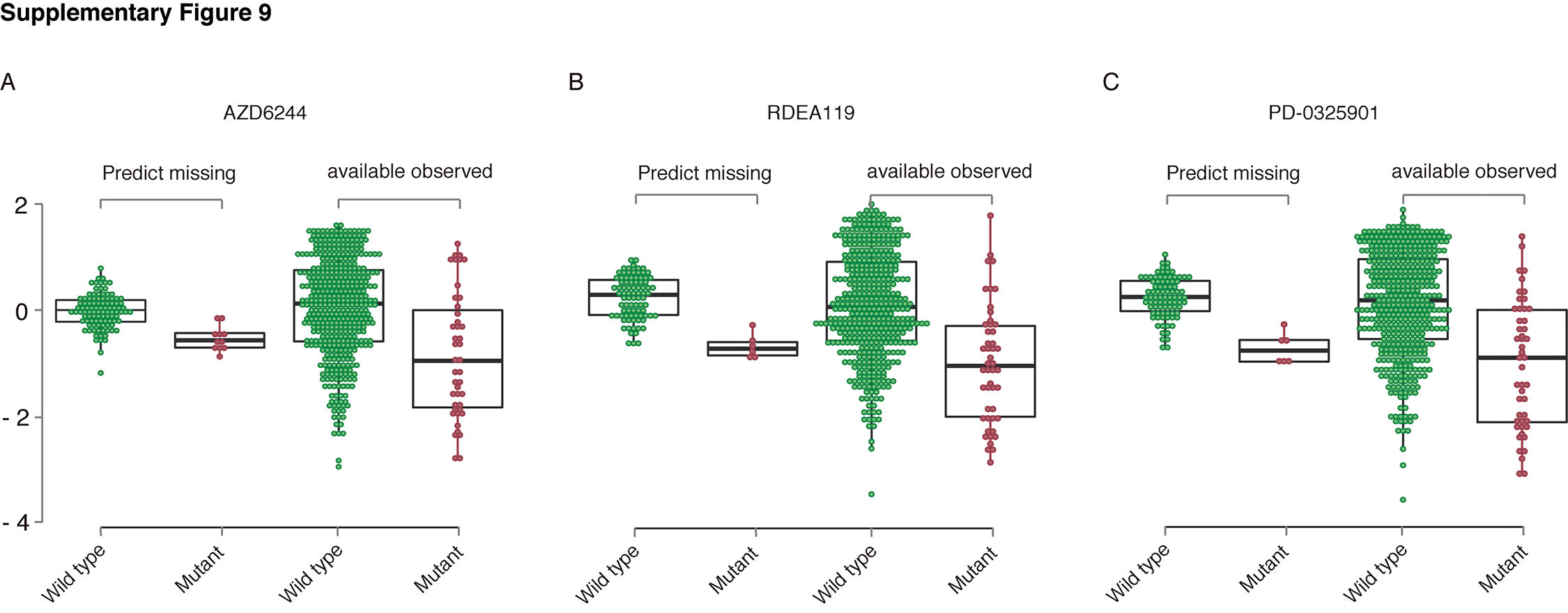

Supplement: S9 Fig — (TIF) [file pcbi.1004498.s009.tif]
